# Arthroscopic Treatment of Septic Arthritis of the Elbow in a 4-Year-Old Girl

**DOI:** 10.1155/2015/853974

**Published:** 2015-12-02

**Authors:** Masashi Koide, Yuichi Tojo, Yoshihiro Hagiwara, Souichi Nakajima, Minoru Tanaka, Masahito Honda, Eiji Itoi

**Affiliations:** ^1^Department of Orthopedic Surgery, Tohoku University School of Medicine, 1-1 Seiryo-machi, Aoba-ku, Sendai 980-8574, Japan; ^2^Department of Orthopedic Surgery, Takeda General Hospital, 3-27 Yamaga-machi, Aizuwakamatsu 965-8585, Japan

## Abstract

Pediatric septic arthritis is uncommon and has been traditionally treated by joint aspiration or open arthrotomy. There are some reports about arthroscopic surgery in pediatric septic arthritis of the knee, hip, and shoulder. However, there is no report for the case of elbow. We report a case of pediatric septic arthritis of elbow treated with arthroscopically with good clinical condition at 3-year follow-up. This paper is based on a report first published in Japanese (Tojo (2012)).

## 1. Introduction

Septic arthritis in children is uncommon. Recent studies reported that the occurrence of pediatric septic arthritis is about 1 to 37 cases in 100,000 in the general population [1, 2]. The lower limbs are especially prone to the disease. Septic arthritis of the hip, knee, and ankle is prevalent in about 85% of all cases [1]. Septic arthritis is considered to be a medical emergency because the articular cartilage is immediately damaged. Unless appropriate diagnosis and treatments are administered, the clinical results can lead to a permanent disability such as osteoarthritis and joint stiffness. The diagnostic and therapeutic delays are the main prognostic factors [3, 4]. These sequelae of pediatric septic arthritis are so devastating that it has been considered that drainage of pus from the affected joint is an essential treatment. Joint aspiration and arthrotomy have been the main surgical interventions for septic arthritis [4].

In the case of adults, arthroscopic surgery has been becoming more popular. This surgery is considered to be effective in septic arthritis because it is minimally invasive with drainage of the joint under full visualization, and we can wash out joint fluid by the amount of fluid irrigation [4, 5]. Owing to this efficacy, arthroscopic surgery is becoming a preferred method also in children affected by septic arthritis of the knee, hip, and shoulder rather than arthrotomy [1–3, 6–8]. However, there is no report for the case of elbows.

This paper reports a case of septic arthritis of the elbow in a four-year-old child treated arthroscopically with good clinical condition at three-year follow-up. This paper is based on a report first published in Japanese [9].

## 2. Case Presentation

**Figure 1:**
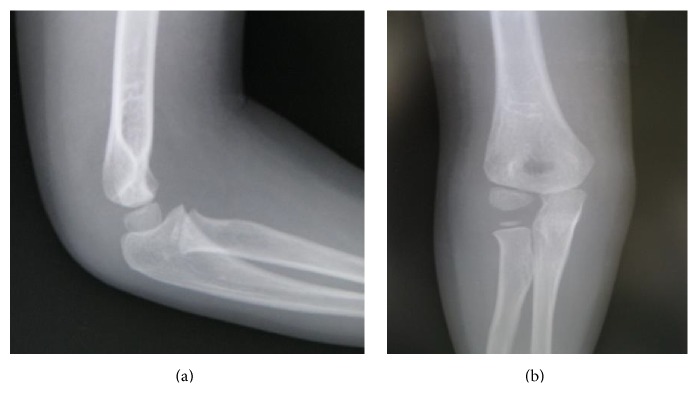
Plain X-rays of the right elbow. There is no sign of osteolysis. (a) Lateral view. (b) Anteroposterior view.

**Figure 2:**
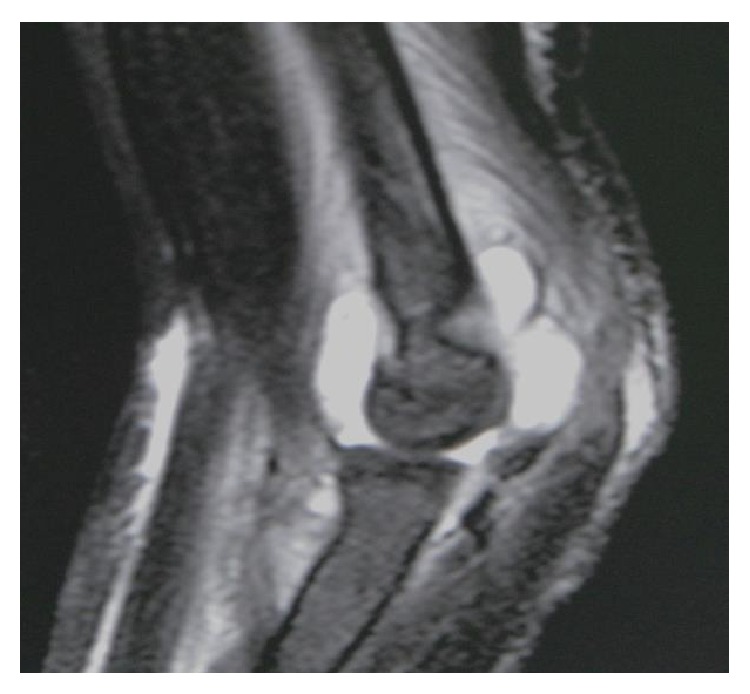
A fat suppressed T2-weighted MRI. A sagittal MRI revealed a presence of joint fluid pooling in the elbow and swelling of soft tissues around the joint.

**Figure 3:**
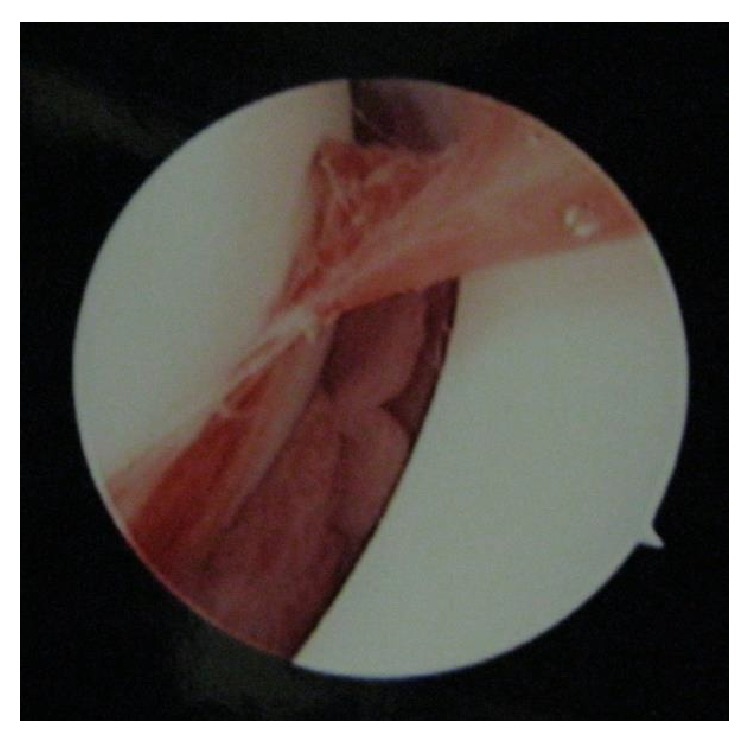
Arthroscopic observation of the right elbow. There was severe synovial proliferation in the joint.

A 4-year-old girl presented to our hospital with fever and right elbow pain. She had no history of trauma. Her past medical history included the DOOR syndrome, which is characterized by mental retardation, sensorineural deafness, and variable seizures but has no immune abnormality. Physical examination revealed redness, swelling, and local heat around the right elbow. Plain X-rays of the right elbow showed swelling of soft tissues without signs of osteolysis (Figure 1). Laboratory studies demonstrated elevated inflammatory markers such as C-reactive protein (CRP) of 8.45 mg/dL and a white cell count of 30100/mm^3^. A total of 3 mL joint aspiration was composed of slightly cloudy synovial fluid. A fat suppressed T2-weighted MRI scan revealed the presence of joint fluid pooling in the elbow joint and swelling of soft tissues (Figure 2). Based on these findings, a diagnosis of septic arthritis of the right elbow was made and the patient was admitted for treatment. She received intravenous cefazolin sodium for 3 days and the examination of the blood test showed a CRP of 4.57 mg/dL and a white cell count of 17000/mm^3^. Because the inflammatory markers still remained higher, the patient underwent surgery of arthroscopic debridement of the right elbow. The surgery was conducted in the same way as the adult one. The patient was in a prone position with the upper limb hanging down on an arm board. An anterolateral portal was created to allow assessment of the joint. The medial portal was established using the outside-in technique under visual guidance. The most important point is that the surgery was conducted by use of a 30° wrist arthroscope to visualize the small pediatric elbow joint. There was much proliferation of synovial bursa in the elbow joint, and debridement was performed under 3000 mL of arthroscopic irrigation (Figure 3). The splint fixation was used for 1 week to protect the elbow joint.

**Figure 4:**
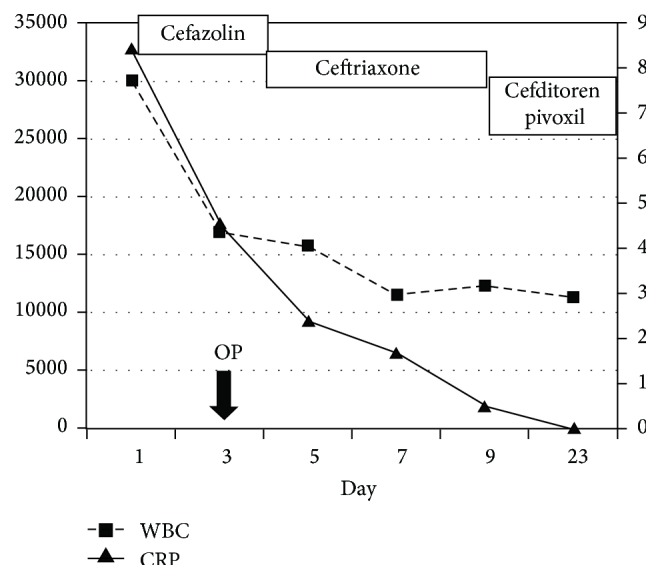
Sequential changes of a CRP and a WBC counts. A CRP was still higher on day 3 and the arthroscopic surgery was performed. After the operation, the CRP level gradually decreased and normalized. OP: operation, CRP: C-reacting protein, and WBC: white blood cell.

On the next day of the surgery, the culture of the first aspiration of the joint revealed* Streptococcus pyogenes.* The antibiotic was changed to ceftriaxone sodium hydrate for 5 days and discharged with cefditoren pivoxil orally, which continued for 13 days (Figure 4). The patient is now three years after surgery and has no osteoarthritis without stiffness of the right elbow.

## 3. Discussion

Pediatric septic arthritis is rare in developed countries. A recent study reported the incidence of it as 1 to 37 in 100,000 cases [1, 2]. However, the situation is different in developing countries. The incidence of pediatric septic arthritis in Malawi is reported as 1 in 5000 cases [10]. The lower limbs are most susceptible to septic arthritis. The occurrence of pediatric septic arthritis is reported as 37% in the hip, 25% in the knee, and 23% in the ankle [1]. Septic arthritis of the elbow is relatively rare, which is 6% of all cases [1]. Septic arthritis in children is a potentially devastating disease that may cause permanent disability or even death. Prompt drainage and washout of the affected joint are advocated for both diagnostic and therapeutic purposes, because the articular cartilage is damaged in the early stage [4]. The functional outcome of the affected joints depends on the time interval between the onset of the symptoms and surgical intervention [3]. However it is sometimes difficult to diagnose because the symptoms and signs are often subtle especially in neonates and young infants [5].

Optimal treatment for a septic arthritis patient requires a combination of medical and surgical interventions. Initial management includes adequate drainage of pus, collection of specimens for culture and antibiotic susceptibility testing, and prompt initiation of appropriate antibiotic therapy [2, 6, 10]. Though earlier surgical intervention is considered to be necessary [1, 4], the indications for surgical drainage of septic joints other than the hip remain controversial [2, 10].

Open arthrotomy was advocated for the standard surgical treatment of septic arthritis especially in the hip, but increasing evidence suggests that there are no recommendations for routine arthrotomy [1, 2, 10]. Pääkkönen and Peltola insisted that the arthrotomy should not be performed after diagnostic aspiration unless the clinical response is poor and CRP levels are increased or remain high [1]. Some authors insisted that aspiration of the joint is a satisfactory method for pediatric septic arthritis [11–13]; however, only an aspiration method may have an insufficient effect on drainage [2, 7].

Arthroscopic drainage of pediatric septic arthritis in the knee, hip, and shoulder has been reported [2–5, 7, 8, 10]. Arthroscopic drainage has many advantages compared with the other surgical procedures. It is less invasive and provides direct and full visualization of the affected joint. Further, it can wash out the joint in large quantities of irrigating fluid under general anesthesia and requires shorter immobilization and hospitalization [1, 3]. In the controlled study of septic pediatric arthritis of hip, El-Sayed reported the shorter hospital stay of the arthroscopic group compared to the arthrotomy group [3]. Further research about pediatric septic arthritis in case of elbow joint is also necessary in the future. The arthroscopic surgery can replace the arthrotomy basically in all cases, but the immediate and complete drainage of the septic joint is mandatory. In our case, the good functional results were achieved by arthroscopic drainage. Arthroscopic drainage of pediatric septic arthritis of the elbow can be a standard surgical treatment like other joints.
